# Correction

**DOI:** 10.5195/jmla.2017.129

**Published:** 2017-01

**VOLUME 104**

**104(4) October, page ii and page 315**

Huffman IR, Martin HJ, Delawska-Elliott B. Creating a library holding group: an approach to large system integration. J Med Libr Assoc. 2016 Oct;104(4):315–8. DOI: http://dx.doi.org/10.3163/1536-5050.104.4.013.

Page ii and page 315: Basia Delawska-Elliott’s name is misspelled in the author byline. The author byline should be:

Isaac R. Huffman, MSLS, AHIP, Heather J. Martin, MISt, AHIP, and Basia Delawska-Elliott, MLIS

